# Nutrition labels’ strengths & weaknesses and strategies for improving their use in Iran: A qualitative study

**DOI:** 10.1371/journal.pone.0241395

**Published:** 2020-10-30

**Authors:** Shirin Seyedhamzeh, Saharnaz Nedjat, Elham Shakibazadeh, Azam Doustmohammadian, Hedayat Hosseini, Ahmadreza Dorosty Motlagh

**Affiliations:** 1 Department of Community Nutrition, School of Nutritional Sciences and Dietetics, Tehran University of Medical Sciences, Tehran, Iran; 2 Students’ Scientific Research Center, Tehran University of Medical Sciences, Tehran, Iran; 3 Department of Epidemiology and Biostatistics, School of Public Health, Tehran University of Medical Sciences, Tehran, Iran; 4 Department of Health Education and Promotion, School of Public Health, Tehran University of Medical Sciences, Tehran, Iran; 5 Gastrointestinal and Liver Diseases Research Center, Iran University of Medical Sciences, Tehran, Iran; 6 Department of Food Sciences & Technology, National Nutrition & Food Technology Research Institute, Shahid Beheshti University of Medical Sciences, Tehran, Iran; Yenepoya Medical College, Yenepoya University, INDIA

## Abstract

**Background:**

This study aimed to explain the strengths and weaknesses of the Traffic light label (TLL) and nutrition facts label (NFL) and the strategies for improving their use in Iran, based on the perspectives of different stakeholders, including mothers, food quality control experts (FQC), nutritionists and food industry experts.

**Methods:**

We conducted 10 Focus Group Discussions (FGDs) with 63 mothers, 10 semi-structured interviews with FQCs, 1 FGD with 6 nutritionists and 1 FGD with 8 food industry experts. To clarify some of the questions that arose from the interviews, the researcher interviewed three policy makers who had sufficient information about the TLL. The discussions and interviews were transcribed verbatim and MAXQDA10 software was used for coding.

**Results:**

The most important findings of this study based on different stakeholders’ perspectives were as follows: mothers believed that nutrition labels reduced the consumption of high-calorie products, although they found the TLL to be easier to understand than the NFL because of its red color. However, their weaknesses were their incompatibility with culture and the lack of trust in the information provided by manufacturers. FQCs pointed out the possibility of changing formulations and the appropriateness of the traffic light for patients, but like mothers, they believed that the labels did not suit the governing culture. Further weaknesses were, misleading the consumer, problems in the colorings reported by different laboratories, and different approaches adopted by regulatory experts. The simplicity of understanding TLL for the general public has been suggested by some nutritionists. Nevertheless, the multiplicity of colors of the TLL was the most important weakness presented by nutritionists and food industry experts and the failure to implement nutrition labels was another issue raised by experts. To improve the use of nutrition labels, notification via media especially television, community education and culture building were suggested by all stakeholders.

**Conclusions:**

The findings of this study underscore the importance of implementing the policy of nutrition labeling in Iran. Mothers and nutritionists believed TLL to be more appropriate for the public to understand, however, FQCs and food industry experts believed that NFL was more suitable in guiding consumers toward healthy food choices. Education and information dissemination via media on interpretive TLL may affect consumer behavior toward food purchases.

## Introduction

Adopting an effective policy to reduce non-communicable diseases (NCDs) is a valuable public health achievement which faces many challenges worldwide. Environmental change and policy-making should be accompanied by programs that motivate people and enable them to choose healthier foods and improve their physical activity [1]. Various policies are being implemented in the world to improve the food pattern, of which food labeling [2, 3], taxes and subsidies [4, 5] are examples of these programs. One of these cost-effective policies is nutrition labeling [6], which contains information that guides consumers toward making better food choices, and may be considered among the most important priorities of preventing NCDs in Iran [7]. Various labeling models have been designed and used so far to reduce the burden of non-communicable diseases like obesity. However, the impact of different nutrition labels on behavior has been inconsistent on healthy food choices [8–10]. Based on the definitions of the ‘Food and Agriculture Organization of the United Nations’ [11] and the ‘Institute of Standards and Industrial Research of Iran’ [12], the nutrition facts sheet is a description of the nutritional facts of a food product designed to inform the consumer. It consists of two parts, nutrients and supplemental nutritional information. In the supplemental nutritional information section food group signs and other pictorial or colored displays can be used, with or without announcing the nutrients.

The use of the nutrition facts label (NFL) on packaged food products became mandatory in Iran by 2014. Owing to the surging trend of obesity and the results of earlier studies on people's lack of attention to such labeling [13–15], policymakers decided to apply interpretive traffic light labels (TLL) on all packaged food products [16]. Thus TLL is now used instead of NFL on all packaged products in Iran. This label has been voluntarily taken up in the UK since 2006 [17]; Sri Lanka and Ecuador are other countries that have implemented this label [18, 19].

To promote healthy food choices the newly adopted TLL in Iran contains information on salt, sugar, fatty acids, trans-fatty acids, calories, as well as portion size. Red, amber and green refer to high, medium and low amount of the aforementioned substances. Using NFL is now optional in Iran, although many factories need to publish NFL as well as TLL because of export.

Food industries opposed the implementation of TLL [16] and specialists, including nutrition and food industry experts were not sure about the impact of TLL on consumers' food choices. One of the reasons of disagreement among experts regarding the variety of label designs is due to different study results, which indicate various factors such as socio-economic status [20], gender [21], nutrition knowledge [22], dietary behavior [23], etc. to be influential. Since legislations regarding packaged products seem challengeable in an obesogenic environment, taking into account the opinions of consumers, FQC experts, experts and policymakers can help find a better solution to improve the status quo and to provide a better option for community health.

The main goal of this study was to explore different aspects of NFL and TLL and to clarify the strengths & weaknesses of NFL and TLL and the strategies that can improve their use from the aforementioned stakeholders' perspectives.

## Materials and methods

This analysis is part of a larger mixed method study that threw light upon the current opinions on existent labels and designed a new type of 'physical activity calorie equivalent label' in Iran to compare its effect with TLL on consumer food choices. A detailed description of the study design has been published previously [24]. The Standards for Reporting Qualitative Research (SRQR) and the Consolidated Criteria for Reporting Research (COREQ) have been reported.

**Fig 1:**
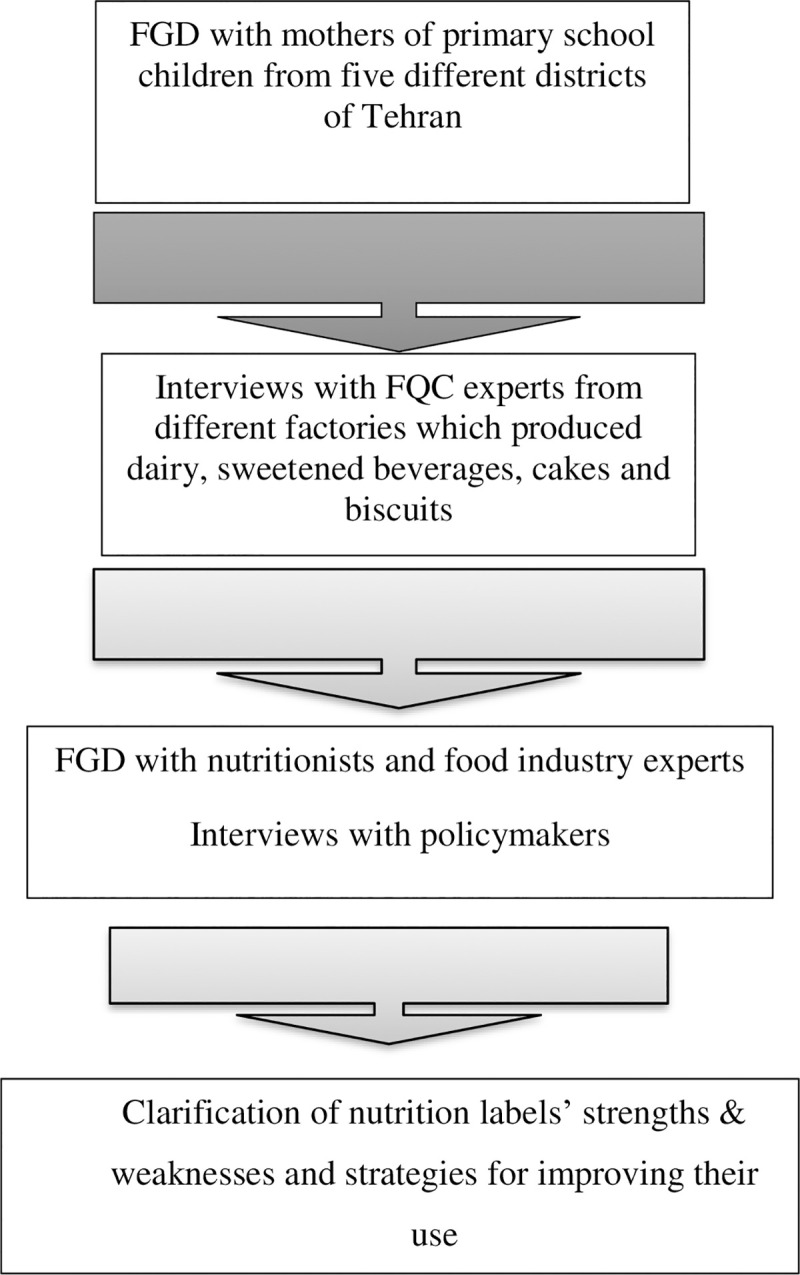
Study design.

Fig 1 shows the design of the present study.

### The strengths and weaknesses of the current NFL and TLL were identified by the following qualitative sub-studies

#### 1. FGDs with mothers as affected stakeholders

*1–1*. *Selection criteria*. Participants were literate mothers of primary school students (6–12 years) who wanted to participate in the study. Mothers who were nutritionists or food industry experts were not included. No limitation to the participating mothers’ age was applied.

*1–2*. *Study design*. FGDs were carried out with different groups of women who were household representatives. A total of 89 participants took part in our FGDs and interviews (Table 1). We intended to apply maximum variation sampling in terms of socio-economic status through school selection. Tehran was divided into five districts: Northern, Southern, Eastern, Western and Central. Then, two elementary schools were randomly selected from each district. In each school, after inviting the mothers to participate in the study, those who were interested in participating in the study were phoned to inform them of the date and time of the FGD. We continued until at least six mothers based on our selection criteria accepted to participate in the study. Hence, our sampling was purposive [25]. We held one FGD in each school and a total of ten FGDs were conducted to achieve data saturation. The FGD was conducted by a researcher who was a nutritionist and had passed a course in qualitative study. She held the FGDs under suitable circumstances at each school and somewhere the participants felt comfortable. Before the meeting, the mothers were asked about the venue, whether it was appropriate for them. The duration of the meeting was estimated in advance so that they could adjust their time if needed. Refreshments were also served. The researcher had a guide for asking the questions and avoided asking leading questions that might influence the participants’ answers. By starting a discussion, the facilitator focused on the key themes prepared in advance. She only guided the discussion and tried to involve all the participants in the FGDs.

**Table 1:** Participants’ characteristics.

**Mothers who participated in the FGDs and their children**		
Mothers’ age* (N = 63)	38.54±5.00	
Children’s age* (N = 63)	9.13±1.44	
Mothers’ educational status	Under high school graduate: 8.2%	
	High school graduate: 36.1%	
	Associate and bachelor degree: 44%	
	Higher than bachelor’s degree: 11.7%	
**FQC experts**	**Education**	**Position in the food industry**
Education	Nutrition (N = 1)	Food quality control manager (N = 1)
	Food industry (N = 7)	Food quality control manager (N = 4)
		Food quality control expert (N = 3)
	Chemistry (N = 2)	Food quality control expert (N = 2)
**Nutritionists and food industry experts**		
Education	PhD in Nutrition (N = 6)	
	PhD in Food Industry (N = 8)	

*Mean ± SD (Standard Deviation).

At first, after a brief introduction the purpose of the study was explained to the mothers and the informed consent form was filled out. Educational status and households' possession of goods were asked through a questionnaire by the investigator. FGDs were conducted with mothers using the question guide in Table 2. All FGDs were recorded during the sessions by a voice recorder and a note-taker. The average duration of the FGD was 1h 45m. Upon showing the mothers some packaged products’ TLL and NFL labels we learned that they were aware of them. Meetings continued until data saturation was reached, meaning no further new comments were added to the earlier ones.

**Table 2:** Questions asked from stakeholders.

**Mothers’ questionnaire (used in the FGDs)**	
1	Suppose you are in a chain store and want to shop. What do you consider when you want to buy packaged foods? Why?
2	Have you ever noticed nutrition labels including the traffic light label or the nutrition facts label? Why?
3	What are the strengths of each of these labels?
4	What are the weaknesses of each of these labels?
5	Do you think it is necessary for food products to have a nutrition label?
6	How we can improve the usage of nutrition labels?
**Semi-structured questionnaire for FQC experts**	
1	What do you think of nutrition labels in general?
2	Do you think it is necessary to utilize nutrition labels? Why?
3	What are the strengths of these labels?
4	What are the weaknesses of these labels?
5	What problems did you face while implementing the mandatory traffic light label?
6	What solutions do you have for solving these problems?
7	Has the new label (TLL) changed formulations? How?
**Nutrition and food industry experts’ questionnaire**	
1	What do you think of the available nutrition labels?
2	Do you think these labels are easy to understand for people? Why?
3	What are the strengths of the existing labels?
4	What are the weaknesses of the existing labels?
5	How we can improve the usage of nutrition labels?

#### 2. Interviews with FQC experts

*2–1*. *Study design*. After holding FGDs with mothers, interviews were conducted with FQC experts. Snowball sampling was used [26]. Participants from famous brands were selected and they in turn introduced other people. Before starting the semi-structured interview, the interviewees were informed about the purpose of the research and the questions in Table 2 were asked.

After obtaining permission, all FGDs and interviews were recorded by a voice recorder. The interviews were recorded by a voice recorder after asking for their permission. Furthermore, participants were assured that the information would only be used for research purposes and would not be accessible to others not in the research team. The average duration of the interview was 45m.

#### 3. FGDs with nutritionists and food industry experts

*3–1*. *Study design*. Sampling method was purposeful and we contacted experts in two universities of Tehran. First, the investigators contacted the Heads of Departments of Nutrition and Food Sciences through official letters and explained the study objective. Further details on the study method and goal of setting up the meetings were described in an email so that other relevant instructors/professors would be informed as well. Thereafter, a day was appointed to hold the focus group discussion (FGD) for the aforementioned instructors/professors.

One FGD with 6 nutritionists and one FGD with 8 food industry experts were conducted to obtain their opinions on the strengths and weaknesses of existing labels. On the day of the meeting, the researchers first described the method and goal of the study and then began to ask questions using the questioner’s guide. The average duration of the interview was 1h 30m.

#### 4. Interviews with policymakers

The researcher interviewed three policy makers who had ample information about the TLL. These policy makers had been introduced by the FQC experts.

### Data analysis

A directed content analysis was applied. Transcripts of all interviews and focus groups were imported into MAXQDA 2010. To analyze the data, the interviews were transcribed ad verbatim on the same day the interviews were held. The information written by the note-taker was added to them afterwards. In order to check the quality of the data collected, half the transcriptions of interviews held with FQC experts and one conducted with a policymaker were emailed to the interviewees (member check). The first researcher read all the transcripts and highlighted aspects of the text and similar information was grouped into codes. The list of codes and their meanings were discussed with the second author. After consensus a codebook was developed by the first author with detailed transcriptions. Furthermore, fifty percent of the transcripts were analyzed by the fourth author and the coding sheets were compared with the first author for inter-coder agreement (90%). Two coders analyzed the information using MAXQDA 2010 [27], and the study team’s qualitative experts checked the analysis results.

Credibility and confirmability of the data were established by prolonged in-depth engagement with the participants. Prolonged engagement with participants in the research environment allowed the researcher to gain their trust and a deeper understanding of their situation. All research details including procedures, actions and decisions were documented for transferability purposes.

#### Ethical approval and consent to participate

This study was approved by Tehran University of Medical Sciences under the registration code 96-03-161-37037 and registered at the Iranian Registry of Clinical Trials [https://irct.ir/] under the code 20181002041201N1. The mothers signed the consent form before the FGDs. A formal letter describing the purpose of the study was sent to the relevant factories or organizations at least one week before interviews with FQC experts and policymakers were conducted, which were conducted upon mutual agreement.

## Results and discussion

For each stakeholder, codes were categorized into strengths, weaknesses and strategies to improve the use of nutrition labels. In the first analysis, we identified 768 codes for the mothers, 163 codes for FQC experts and 90 codes for experts. Upon frequent analysis and exclusion of similar codes a total of 75 codes including 15 codes for strengths, 38 codes for weaknesses and 22 codes for strategies to improve the use of nutrition labels from all stakeholders remained.

Given the importance of different stakeholders' perspectives, the strengths, weaknesses and strategies of each group of stakeholders was described separately.

**Fig 2:**
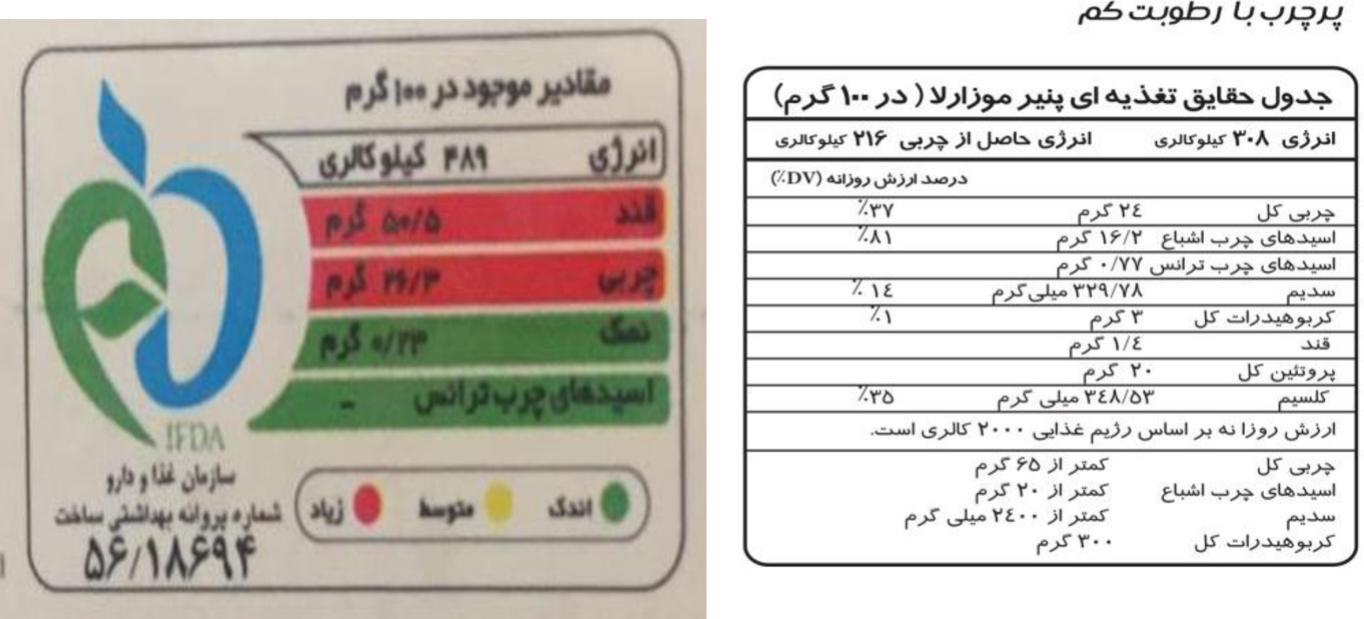
(a) Traffic Light Label (TLL) nutrient content in 100 grams of chocolate (energy: 489 Kcal, sugar: 50.5 g (red), fat: 26.3 g (red), salt 0.23 g (green)) green: low, amber: medium and red: high, (b) Nutrition Facts Label (NFL) nutrition fact panel in 100 grams of mozzarella cheese (energy: 308 Kcal, total fat: 24g and %Daily Value(DV): 37%, saturated fatty acid: 16.2 g and %DV: 81%, trans fatty acid: 0.77 g, sodium: 329.78 and %DV: 14%, total carbohydrate: 3g and %DV: 1%, sugar: 1.4g, total protein: 20 g, calcium: 348.53 mg and %DV: 35%).

Fig 2 shows mandatory TLL and optional NFL in Iran.

### FGD with mothers as affected stakeholders

Six codes for strengths, 8 codes for weaknesses and 9 codes for strategies were identified. The strengths, weaknesses and strategies for improving the use of nutrition labels (both NFL and TLL) are shown in Table 3. Each theme is discussed along with its codes (bold fonts) to provide further details.

**Table 3:** Strengths, weaknesses and strategies for improving the use of nutrition labels, based on mothers’ viewpoints.

Themes	Codes	Number (frequency %)
**Strengths**	1. Easy to understand and prominent red light of the TLL	18 (35%)
	2. Healthier food choices at the point of purchase	12 (23%)
	3. Reduction in purchase and consumption of high calorie products	12(23%)
	4. Increased thoughtfulness following diseases	8 (15%)
	5. Raised awareness about energy	2 (4%)
**Weaknesses**	Labels’ small sizes, fonts and inappropriate location in packaging	14 (26%)
	1. Ambiguous and high amount of information provided	11 (21%)
	2. Mistrust in labels and information provided by manufacturers	9 (17%)
	3. Nutrition labels do not conform to our culture	6 (11%)
	4. Unfamiliarity with nutrition labels	5 (9%)
	5. Unattractive labels	3 (5%)
	6. Over-sensitizing people	2 (3%)
	7. No impact on food choice for some people	2(3%)
**Strategies**	1. Notification via mass media	15 (32%)
	2. Community education	13 (28%)
	3. Improving eligibility, simplicity and location in packaging	6 (13%)
	4. Publication of monitoring and evaluation results	4 (8%)
	5. Culture-building	4 (8%)
	6. Encouraging factories to produce healthy products	2 (4%)
	7. Nutritional information on store shelves	1 (2%)
	8. Coloring based on products’ quality	1 (2%)
	9. Including consumer age groups	1 (2%)

#### Strengths

Nutrition labels result in **healthier food choices at the point of purchase** or **reduction in purchase and consumption of high calorie products.** High calorie and fat content are the most important reasons for reducing consumption or not choosing certain food items. Mothers' **awareness about energy has increased;** they want to know the amount of energy each product has.

It seems that maternal weight or child health status may impact their food choices. Some mothers claimed that their **attentiveness** regarding nutrition labels **increased following diseases**. If a mother or a member of her family had suffered from diseases like diabetes, hypertension, obesity and cancer, she had paid more attention to nutrition labels afterwards. *"Unfortunately*, *I personally don`t care about my health as long as I am healthy*. *Health considerations begin as soon as something goes wrong” (43-year-old mother)*.

The **easy to understand and prominent red light of the TLL** were specifically addressed. For both adults and children, the TLL colors make it easier to understand than the NFL; since consumers do not need to pay much attention to read or interpret it. *"It will convey the message quickly*, *that you can make a better choice by choosing green colored label products" (29-year-old mother)*.

#### Weaknesses

**Mistrust in labels and information provided by manufacturers** is an important concern of the mothers regarding the food industry. Certain unpleasant and/or inaccurate news in the media has made people distrustful toward the information delivered to them, including nutrition labels on products. *"Maybe these things were not true in the first place… People probably think these labels are not true and so can’t be trusted "(33-year-old mother)*.

Nutrition labels in Iran have been designed using other developed countries’ experiences. Thus, according to the mothers, these labels do **not conform to our culture**. "*As usual*, *we Iranians give great importance to hospitality*. *We usually try to get the best when we have guests*. *For example*, *if we always consume low-fat dairy*, *we will buy high-fat dairy for our guests to make the food taste more delicious*" (31-year old mother). Another 39-year old mother said: "*For example*, *we do not consume sugar at all*. *We use honey*, *honey that comes from natural resources*, *not factories*, *based on what we are aware of*. *The degree of this awareness varies among people*, *but based on the concerns that exist*, *many people do not pay attention to these nutrition labels and do not have time for them*. *For example*, *my spouse first paid a lot of attention to expiry dates*. *Once we bought a drink and saw that it was past its expiry date*, *we drank it anyway and didn’t die*! *These are cultural matters*. *In the old days we had more trust; I would go to a shop and buy something and believe that it was healthier*."

The physical characteristics of these labels were also discussed, and **labels’ small sizes, fonts and inappropriate location in packaging** were pointed out. Usually, nutrition labels are at the back of the package and the participants argued that they needed to spend a lot of time to find and read them. Another weakness the mothers cited was the **ambiguous and high amount of information provided**; portion size in grams or serving size, as well as carbohydrate and trans-fatty acids cause confusion among consumers.

Another frequent complaint was **the unattractiveness of labels** due to **small sizes** and **fonts**, and **inappropriate locations** that make the appearance of nutrition labels unpleasant and useless.

In some mothers’ opinions, nutrition labels not only **over-sensitize people** but can have the opposite effect, and have no impact on food choices. *"Nutrition labels can deviate us from healthy food choices by over-sensitizing us" (39-year-old mother)*.

Some mothers claimed that their food choice would not change even if they knew the high amount of calorie, sugar and fat. *"I know that I and my family are obese but I do not want to say no to my child when he wants to eat cake or chips*. *He is too young and should eat whatever he wants*. *These labels don’t matter to me*, *even if they improve" (42-year-old mother)*.

Although nutrition labels have been on food products for many years, people are still **unfamiliar with them**. Several mothers specifically mentioned the labeling of electronic supplies, which has been widely supported in television advertisements. *"It's been a while that power consumption information is shown in the middle of TV commercials in forms of A and B labels*. *When such form of broadcast increases*, *I feel that most people who want to buy appliances pay attention to it*. *My 80-year-old mother has learned it*. *My 6-year-old son too has learnt that when he sees a short flash it means energy consumption is low" (40-year-old mother)*.

However, mothers claimed they did not have sufficient nutritional knowledge to use the other information provided in the TLL, e.g. trans-fatty acids.

#### TLL versus NFL

**Upon comparing TLL and NFL, many of the mothers stated that** TLL was more eye-catching than NFL. *"The traffic light label is better because it draws more attention at a glance*, *for example we know green is good*, *and red is bad and you can easily make out its’ meaning" (38-year-old mother)*. Based on their opinion, traffic light labels are easier for children to understand and easier to recognize on packaged products.

*1*. *Strategies to improve the use of nutrition labels*. **Notification via media** and **community education** were the most common strategies addressed by mothers. One way to increase the use of nutrition labels is to inform and educate the community. However, most of the mothers did not know how to use TLL in their food purchases. According to some mothers, participation in this study and involving them increased their awareness about nutrition labels. Education can be delivered at community level in centers such as kindergartens, schools and health services centers. These programs will increase consumers' awareness of labels and will teach them how to interpret labels while choosing food products.

Another strategy mentioned was **culture-building**. The use of nutrition labels at the point of purchase requires cultural infrastructure and this can be achieved by raising awareness first. *"Culture is the backbone of all problems*, *not only in this regard*, *but in every field*. *When we have information about something*, *we certainly use it*. *Who would not want to use the best things*?*" (33-year-old mother*).

Participants reflected that **eligible** and **front of pack** nutrition labels would affect consumers' behavior in purchasing food items. However, in many products, nutrition labels are at the back of the pack. *"We sometimes buy some goods*, *and after eating them*, *see how many calories they have*. *We then decide not to purchase them anymore" (41-year-old mother)*.

**Publication of monitoring and evaluation results** by responsible authorities would influence consumers’ trust in the food industry. The evaluation process should be communicated with people through the media, especially through television to ensure that the information on nutrition labels is genuine and reliable. **Encouraging factories to produce healthy products** with regards to sugar, salt and fat content would help people make better choices. *"The authorities can award manufacturers who produce healthy products; they can even hold competitions to encourage the manufacture of healthy items" (35-years-old mother)*.

According to some mothers, **nutritional information** can be displayed **on store shelves** in chain stores, although its feasibility needs more assessment.

For years, dairy factories used coloring in their packages based on the degree of fat content, which was easily understood and used by consumers. For example, a green milk bottle cap indicated a low-fat product in a specific brand. Some mothers suggested that **coloring based on the products’ quality** were based on their nutrition content and that they had recognized them in their purchases. However, different brands have defined different colors for categorization and it is not easy to integrate packaging and apply it to other products. The last recommended strategy was that factories could package their products in different sizes based on different **age groups**. One of the mothers who had an experience with a foreign product said *"the smaller chocolate cake was for 12-year-old children and the other was for 12 to 24-year-olds …*.*"*

#### Mothers as household representatives

The mothers believed that TLL was more comprehensible and informative than NFL in providing information on calories, fat and sugar. They also mentioned that if their children were educated on how to use the TLL, their food choices would be affected by the colors. These results are consistent with previous investigations indicating that TLL is easy to understand for consumers [19]; furthermore, a significant decrease in purchasing unhealthy foods was observed among children [28].

In Iran, the TLL was replaced by the NFL to decrease the growing trend of NCDs [16]. In high income countries such as England the positive effect of TLL has been observed [8] and a recent study in Canada (using a realistic scenario) estimated that TLL could have positive effects on NCD outcomes [29]. According to our findings, people who suffer from NCDs pay more attention to nutrition labels. Therefore, to achieve the best results of using nutrition labels during food purchases, it is necessary to educate and create awareness among consumers [30, 31].

### Interviews with FQC experts

The strengths and weaknesses of nutrition labels as expressed by FQC experts are presented in Table 4.

**Table 4:** Strengths, weaknesses and strategies for improving the use of nutrition labels, based on FQC experts’ viewpoints.

Themes	Codes	Number (frequency %)
Strengths	1. Similarity to foreign product labels†	6 (33%)
	2. Suitable for people with nutritional knowledge†	3 (17%)
	3. Colorful*	3 (17%)
	4. Useful for patients*	2 (11%)
	5. Suitable for the public*	2 (11%)
	6. Possibility of changing the formulation*	2 (11%)
Weaknesses	1. Failure of authorities to assess factories’ claims*	7 (14%)
	2. Different approaches of regulatory experts	7 (14%)
	3.Incompatibility of nutrition labels with public culture	5 (10%)
	4. Inconsistency between policymakers	4 (8%)
	5. Misleading*	4 (8%)
	6. Consequences of the red color for manufacturers*	4 (8%)
	7. Different coloring responses from different laboratories*	3 (6%)
	8. Printing TLL and NFL on export products	3 (6%)
	9. Possibility of fraud*	2 (4%)
	10. Focusing on some nutrients*	2 (4%)
	11. Impossible to reformulate	2 (4%)
	12. Financial burden*	2 (4%)
	13. Potentially harmful substances like preservatives	1 (2%)
	14. Weight definitions*	1 (2%)
	Small packaging	1 (2%)
	15. High amount of information and small sizes of labels	1 (2%)
	16. Not taking into account different age groups in nutrition labeling	1 (2%)
Strategies	1. Collaboration with manufacturers	8 (28%)
	2. Notification via media	6 (21%)
	3. Institutionalizing the utilization of nutrition labels	6 (21%)
	4. Community education (especially patients with NCDs)	3 (10%)
	5. Coloring based on product quality	3 (10%)
	6. Taking into account different age groups in labeling	2 (6%)
	7. Defining barcodes	1 (3%)

* Specific to TLL.

† Specific to NFL.

#### Strengths

According to FQC experts, the first and foremost strength of TLL is its **colorful** nature; due to its well-known colors it is **suitable for the public**. The simplicity of TLL makes it suitable for all audiences as they do not need to spend lots of time on interpreting what the label means. Furthermore, it is **useful for patients** with NCDs who want to know which products are more appropriate. Another strength of the TLL is the **possibility of changing the formulation** of products. *"Sometimes formulations change*. *For example*, *the amount of salt was at the amber borderline; then we cut down a little salt and it turned to green" (Food industry expert)*.

Interviewees mentioned that NFL is **suitable for people who have nutrition knowledge**. However, the **similarity of NFL to foreign product labels** makes it more suitable than TLL. *"Why are we trying to reinvent the wheel*? *There is an FDA standard table that mentions all the specifications of serving*, *which the entire world follows" (FQC expert)*.

In general, all interviewees were dissatisfied with the TLL and considered it as a serious production problem. The weaknesses they mentioned mostly lay in the TLL, as they considered NFL as the standard nutrition label format.

#### Weaknesses

**Incompatibility of nutrition labels with people's culture** was the most commonly mentioned weakness of nutrition labels. *"As long as a behavior hasn’t been institutionalized*, *we certainly won't see the desired response among the community*. *People should be educated about nutrition labels through media; they should be aware of what they are consuming and what to choose*. *However*, *in our setting*, *we only force manufacturers to apply labels" (FQC expert)*.

One of the main reasons FQC experts oppose the TLL is its ‘red light’. They think that if consumers see the red color in a product, they will not buy it. Thus, this type of label has **consequences for manufacturers**. *"In my opinion*, *if they increase people's awareness of the TLL*, *it will have consequences for the producers and no-one will buy the products colored red anymore" (FQC expert)*.

The next weakness cited was the **financial burden** of TLL. Changes in the structures of labels and defining new labels with specific characteristics impose costs on manufacturers. Some interviewees said that they had to change the whole design of their labels and some others had problems in printing of different colors. For example, after printing a label the amber turned out very similar to red.

Nutrition labels **do not take into account different age groups**. According to one FQC expert: *"Every product is intended for a particular age group*, *when you change the formulation*, *you may lose your popularity and consumers" (FQC expert)*.

One of the goals of nutrition labels is to encourage manufacturers to improve their formulation. However, it is **impossible to reformulate** some products because of their nature. *"There is nothing we can do about biscuits to make their labels ‘green’ (FQC expert)*.

**Printing TLL and NFL on export products** in accordance with Codex Alimentarius principles [11] and Iran’s FDA [12] have resulted in **high amounts of information in nutrition labels and their small sizes**. Some interviewees also reported that TLL guidelines were not clear in some cases; e.g. their **weight definitions** and in products of **small packaging**. Ambiguous weight definitions particularly became problematic for FQC experts in defining weights in labeling. Participants believed that TLL was **misleading** consumers. *"[As a consumer] I need to know the amount of energy*. *I need to know the serving sizes*. *[For example] I'm overweight*, *or I'm sick*, *but I know nothing [about nutritional values]*. *This is not helpful*. *For example*, *what does the red color mean [in the TLL]*? *What does the orange mean*? *It is a misleading label [TLL] (FQC expert)*.

Nutrition labels **focus on certain nutrients** that might influence consumers’ behavior, whilst **potentially harmful substances like preservatives** are not shown. "*There is no need for all products (for example tomato paste) to have a TLL; to avoid receiving the red color manufacturers are encouraged to replace the ingredients with preservatives*" *(FQC expert)*. This is a major issue for most FQC experts. They do not want their nutrition labels to include ingredients that have side effects. Thus, they replace, for example, salt with chemical preservatives–the former of which might be more important for consumers to stay away from. One of the interviewees underlined that TLL was threatening the food industry because it provoked the **possibility of fraud** in products with high amounts of fat, sugar or salt, as FQC experts would be inclined towards altering their labels’ colors.

For FQC experts, another problem with TLL implementation was laboratory results. Some FQC experts showed the interviewer documents relating to a sample that had received **different coloring responses from different laboratories**. Although these laboratories are introduced by the Ministry of Health and Medical Education (MOHME), their responses do not match each other in some cases.

There was controversy regarding MOHME’s assessment results too. Some FQC experts claimed **supervisors failed to assess the factories’ claims on TLL**; while others approved MOHME’s monitoring by presenting certain documents to the interviewer. Nevertheless, **regulatory experts’ different approaches** were another issue in TLL evaluation. According to most interviewees, the experts’ approaches toward assessment differed and were heterogeneous. *"Law enforcement is executed differently by different experts*. *Some are very strict in adherence to their guidelines and some others are more lenient" (FQC expert)*.

The last weakness mentioned by FQC experts was **inconsistency between policymakers**. One of the interviewees mentioned that every product has its own physical and chemical characteristics which are defined by ISIRI (Institute of Standards and Industrial Research of Iran). *"For example*, *fruit juice has a specific amount of sugar in it*. *If we put less sugar we get punished by Iran’s ‘Suspending Organization’ for using less material" (FQC expert)*.

#### TLL versus NFL

Based on the FQC experts’ opinions, NFL is better than TLL for consumer use. Although the FQC experts all agreed on healthy product development, the lack of incentives and lengthy procedures of manufacturing new goods create difficulties for manufacturers. Moreover, the TLL label is mandatory for all products (except products like oil and sugar) and in particular for sweet products such as cakes, biscuits and juice. The aforementioned products inevitably receive a red label for their sugar content, but producers cannot reduce their sugar contents easily because of consumers’ preferences.

#### Strategies for improving the use of nutrition labels

Similar to the mothers, FQC experts believed that **community education** and **notification via media** would help consumers and especially patients with NCDs to use nutrition labels in their purchases.

The FQC experts believed that using nutrition labels required **culture-building**. **Coloring based on product quality** and **taking into account age groups in labeling** were other strategies of improving the use of nutrition labels. For example, a biscuit might not be suitable for a diabetic patient but it is not harmful to a child.

Encouraging **collaboration with manufacturers** was mentioned as an effective strategy: *"The policymakers should have educated us and for example*, *delivered a list of recommendations to us*. *Then we would’ve known if something went wrong*. *However*, *instead of collaboration*, *they’re just tussling with us" (FQC expert)*.

One FQC expert thought **defining barcodes** could prevent high volumes of printing and was an appropriate strategy for people who wanted to know more about the nutrition facts of a product in their purchases.

#### FQC experts as executive representatives

The FQC experts’ side of the story was completely different from that of the mothers. They were interested in NFL and opposed the TLL as they found it a barrier toward selling their products. They were worried about the consequences of TLL presenting the red color on food items’ packages. This finding is consistent with the study conducted by Corvalán et al. [32]. Here we must add that there were no documents from the factories indicating the increased returns of their high-fat or high-calorie products. The main problem with the FQC experts' opinions was that they had unrealistic beliefs and fear regarding the TLL and in particular with their front-of-pack nature. They thought that TLL would limit the purchase of their high-fat or high-calorie products, so most of them changed its location from the front to the back of the pack and this strategy might not help improve consumers' behavior [33]. To overcome their current problems, individual assessments ought to be avoided and a universal monitoring system based on a predefined checklist should be implemented. The effective involvement of FQC experts as the executive body is required in policymaking to ensure the success of law enforcement.

### FGDs with nutrition and food industry experts

The strengths & weaknesses of nutrition labels and the strategies for improving their use are presented in Table 5.

**Table 5:** Strengths, weaknesses and strategies for improving the use of nutrition labels, based on nutritionists and food industry experts’ opinions.

Themes	Codes	Number (frequency %)
Strengths	1. TLL is easier to understand than NFL	4 (44%)
	2. NFL is easier for people with nutritional knowledge	3 (33%)
	3. Useful for some people	1 (11%)
	4. Importance of food choices*	1 (11%)
Weaknesses	1. Nature of the food product*	7 (14%)
	2. Multiple colors on a label*	6 (12%)
	3. Failure to implement correctly*	6 (12%)
	4. Different weight definitions*	5 (10%)
	5. Inadequate implementation†	5 (10%)
	6. Lack of supervision*	4 (8%)
	7. Misleading consumers*	3 (6%)
	8. Unspecified ranges*	3 (6%)
	9. Defining colors *	3 (6%)
	10. Controversies over fat*	2 (4%)
	11. Mismatching nutritional knowledge*	2 (4%)
	12. Incomplete list†	2 (4%)
	13. Ignoring manufacturers' problems and their facilities	2 (4%)
Strategies	1. Notification via media	4 (27%)
	2. Community education	3 (20%)
	3. Brief and useful information	3 (20%)
	4. Culture-building	2 (13%)
	5. Coloring based on product quality	1 (6%)
	6. Taking into account different age groups in labeling	1 (6%)
	7. Following international standards	1 (6%)

* Specific to TLL.

† Specific to NFL.

#### Strengths

According to the experts’ opinion, the proper cultural infrastructure for the use of nutrition labels has not been established in the country yet. Thus, these labels are **useful for some people.** *"True*, *at first people didn't pay attention but some people did*. *Its existence is better than its nonexistence" (Nutritionist)*. Furthermore, the change in mandating labeling from NFL to TLL shows that policymakers have recognized the **importance of food choices** because of their impact on disease. *"I think this is the first time the Food and Drug Administration is admitting that people's diet has affected their health*. *Well*, *when they came up with the idea*, *this was a step forward; they understood the importance of salt*, *fat*, *sugar and trans-fatty acids and took advantage of nutritionists`knowledge" (Nutritionist)*.

Most of the nutritionists believed that **TLL was easier to understand than NFL** for most people, however, all the food industry experts thought **NFL was easier than TLL to understand for people with nutritional knowledge**. In general, food industry experts disregarded the TLL and did not believe it had any strength and considered the NFL to be a standard food label. They believed that people did not read the numbers in the colors and that could make them choose unhealthy products.

#### Weaknesses

Participants reported that TLL was meaningless because of its **multiple colors.** *"There may be three greens in one product and one red*, *but that red one is more important than the other three" (Nutritionist)*. Thus, according to the experts, different colors were **misleading consumers** in making better food choices.

**Controversies over fat** were mentioned in both FGDs as another weakness of TLL. *"You should be careful not to limit the consumption of useful products; the difference between 3% and 2*.*5% milk is half percent for a consumer who drinks whole fat milk*. *However*, *reducing half percent fat changes the fat label color from red to amber or from amber to green*. *That doesn't make sense*, *but at times the consumer decides not to consume dairy at all*!*" (Food industry expert)*. In addition, inconsistency between NFL and dairy products' content was cited. *"Much of the information provided in NFLs is wrong*. *For example*, *the label of a dairy product with 2*.*5 or 3 percent fat is written as ‘low fat‴ (Nutritionist)*. **Lack of supervision** was also discussed in the nutritionists’ session. *“The FDA has set guidelines defining criteria for low fat products*. *Here (Iran)*, *we do not have that kind of supervision” (Nutritionist)*.

Most participants believed that TLL should take into account the **nature of the food product** *"Jam and fruit juice both get red for their sugar content*. *However*, *they are totally different" (Nutritionist)*. **Unspecified ranges** were cited as another weakness of TLL. *"In my opinion*, *the red in the TLL means that that product should not be consumed*, *however*, *it does not imply such a thing to the consumer*. *For example*, *a dairy product ranging with 1*.*5 percent and higher fat all receive the red label*, *but the difference between them may be huge" (Food industry expert)*.

In both FGDs, some participants expressed that the **failure to implement nutrition labels correctly led to TLL color definitions** based on factory experts’ manual calculations and not on the laboratories’ analysis results. *“The nature of the TLL is appropriate*, *however*, *because of opposition from FQC experts it did not work out well*, *as all producers of junk food should define their labels with red*!*” (Nutritionist)*. **Different weight definitions** were cited as another TLL weakness. “*For example*, *one product gets the red color for a nutrient in 100 grams and the other for 50 grams; consumers become confused” (Food industry expert)*.

Specifically, **incompleteness of the list** and **inadequate implementation** of NFL were addressed by the participants, as NFL was mandatory before TLL but the evidence did not show significant effects on consumers’ behavior. *“Everyone decided to write whatever s/he desired on the NFL label*. *It was mandatory*, *but was not implemented correctly” (Nutritionist)*.

Another weakness mentioned by food industry experts was that **manufacturers' problems and their facilities–**such as printing and label size- **were ignored** by policymakers.

#### Strategies for improving the use of nutrition labels

**Notification via media**, **community education**, **culture-building**, **coloring based on product quality** and **taking into account different age groups** were commonly expressed by all the stakeholders.

**Following global standards** and providing **brief and useful information** were specifically addressed by nutritionists and food industry experts.

**Coloring based on product quality** instead of using TLL was mentioned by some nutritionists. *"I think every product should show its quality on its packaging*. *Each of them must have an instruction to show its quality and level of healthiness*. *For example*, *how much sugar should a jam have to consider it healthy*? *Jam is supposed to be sweet*, *so getting the red color for the sugar in it does not make sense to consumers" (Nutritionist)*.

**Provision of Brief and useful information** was cited as an effective strategy to motivate and prevent confusion among consumers. *"In my opinion*, *the less information they contain*, *the more effective they would be" (Food industry expert)*.

#### Nutritionists and food industry experts

According to nutritionists, the use of TLL has been a step forward in promoting community health, and its implementation reflects policymakers' attitudes toward improving people's nutritional status by preventing NCDs.

However, food industry experts were even more opposed to the TLL than the FQC experts, and it seems that they had passed on their views to the latter as a result of their communications or lobbies. Thus, it might be useful to strengthen the link between policymakers and food industry experts to take into account their opinions in the industrial implementation as well. Their opposition stemmed from certain controversies regarding the TLL. A systematic review and meta-analysis reported that TLL is marginally more effective in increasing the selection of healthier options [34].

Another concern of food industry experts was that dairy fats get the red color in whole-fat products, whereas, the amount of fat in creamy products are much more than the latter, but they receive the same color. Consumption of dairy products per capita in Iran is lower than the average global intake [35] and in the experts’ opinions, dairy fat should be excluded from TLL coloring given the recent findings [36]. Following the regulation on whole fat milk, several meetings were held to decide how to label dairies, and given the potential consequences of further reduction in dairy consumption and its related scientific evidence [37], whole milk acquired green for all its nutritional information, including fat.

According to policymakers, the implementation of the TLL has been a difficult task and, the enforcement of TLL on all products despite its difficulties is a significant approach. Nevertheless, due to poor advocacy, it has been poorly advertised by the IRIB. Although, in the policymakers’ opinion, the main mission of the media, and especially television, is to promote public health, and they do not have any funding issues, especially in the field of community-based health education.

### Interviews with policy makers

Some questions were asked separately to clarify the questions that arose from the interviews. These were about implementation, FDA guidelines, controversies surrounding monitoring, dairy fats, nutritional labeling on all food products and lack of education via media.

The first issue in the policymakers’ opinions was failure in correctly implementing the TLL. The aim of mandating TLL was to improve peoples’ awareness about nutrition labels. *"The more we can inform people*, *the more it is in our best interest*. *There is a supply and demand*, *and people can take care of themselves" (Policymaker)*. However, the regulation of TLL on every product was strictly implemented and policymakers wanted to ratify this label. *"For some reason*, *some FQC experts disagreed*. *Some did not know what was going on; their unions were required to implement labeling*. *Six months later*, *the tribunal announced that the funds had not cooperated*, *and the minister was forced to do so by the following month*. *The technical staff did some calculations*. *Some did well*, *however others did not know how to calculate the labels’ formulations*. *They did not want to get red for a product*, *so they tried to calculate in such a way to get green or amber*. *Some industries*, *such as the dairy industry*, *were hit hard by the fact that their fat was three percent; people didn't buy this milk*, *so there was some opposition as to what to do" (Policymaker)*. Before implementation, a pilot study had not been conducted to assess the effectiveness of this policy and could be considered as another reason of failure of its implementation.

The second issue was the TLL guideline by Iran’s FDA that clearly specifies the font size, location and other information to be displayed on the label; some factories do not follow these instructions. However, one of the barriers of the guideline is its weight definition, which does not precisely mention how to define weights for every product.

The third issue was the controversy surrounding monitoring. Policymakers presented documents indicating that monitoring is being done and has its own guideline. Nevertheless, different individuals might have evaluated labels differently given they had been trained differently.

The next issue was dairy fats. The interviewer recognized that there was an ambiguity about dairy fat among policymakers. Besides the lack of evidence about dairy fat, the palm oil scam had worsened the labeling situation. Thus, in order to prevent the decrease of milk intake and prevent factories’ potential loss, an exception was made for whole milk and factories were allowed to mention this type of fat with green color.

The fourth issue was the nutritional labeling of all food products. According to nutritionists and food industry experts, the nutrition label is not necessary for all food products. However, policymakers believed that every product should have a nutrition label.

The last issue was lack of education via media. MOHME’s budget limitations led to the failure of community education. *"We’ve forgotten about the target group*. *We check it out at the factory*. *We’re not concerned with whether or not it’s consumed afterwards; doesn't matter to us anymore*. *Even if it does matter we can't do anything about it because we don't have the means; our only tool is the media" (Policymaker)*. On the other hand, some were of the opinion that there is no need for a budget to improve public health by raising awareness. *“The main mission of the IRIB (Islamic Republic of Iran Broadcasting) is to improve public health and raise public awareness*, *so we should not allocate budget to this purpose” (Policymaker)*.

Based on our results, most of the mothers’ viewpoints were in line with those of the nutritionists; and the majority of the quality control experts’ opinions were consistent with those of food industry experts. The similarities between the mothers and nutritionists' opinions and between the FQC and food industry experts may have resulted from communications between either pairs of stakeholders. All the stakeholders unanimously believed that neither nutrition labels had been properly implemented. TLL was carried out voluntarily from 2012 to 2014 with the aim of reducing the burden of non-communicable diseases in Iran [16], but after it was not welcomed by the producers, it became mandatory to include it on all products by order of the then Minister. This prompted manufacturers to produce the label and affix it to their products in a short period of time. However, due to their resistance, these labels were printed on the back of the packages instead of the front and were illegible, which was practically against the instructions of the Food and Drug Administration in Iran. Inadequate oversight by evaluators and reference lab errors in calculating nutrient information on labels also led to discrepancies in different products of the same type but manufactured by different factories. Moreover, inadequate information and lack of education of consumers through popular media such as television led to the lack of awareness of the main audience of this policy, namely the society.

Based on our findings, although interpretive labels like the traffic light label are expected to be easily understood given their simplicity, it seems that even this type of labeling needs to be taught to the community.

To determine the effectiveness of this policy in reducing NCDs, mandatory labeling in high-income countries such as England and other European countries [37] and in middle-income countries such as Iran and Ecuador [38] need to be reviewed effectively over time.

## Policy implications

The results of this study can be used by policymakers to elucidate the viewpoints of mothers as household representatives, FQC experts as executive representatives, and nutritionists and food industry experts as scientific representatives on the existing labels. A number of recommendations for policymakers are presented for future interventions: 1) conducting a pilot study to provide sufficient evidence to the policymaker to achieve the desirable outcomes, 2) establishing an appropriate infrastructure in the community through education (culture-building), 3) establishing a specialized workgroup including policymakers, nutritionists, food industry experts, industry representatives, the media and NGOs before implementing a policy.

## Limitations and strengths

The present study has a particular set of limitations and strengths. The strengths and weaknesses of nutrition labels and strategies for promoting their use have been broadly outlined.

FQC experts might have given misleading answers due to conflicts of interest. However, their responses were mostly confirmed by the mothers, experts and policy makers' answers. Yet, despite the problems raised by them, such as the lack of transparency of instructions for printing the TLL, upon investigation, the researcher learned that this problem had been solved by policymakers.

The number of policymakers interviewed was small, but we found it unnecessary to interview any further policymakers as those interviewed had already answered all our questions and had thrown light on the ambiguous issues.

## Conclusions

The main challenge of implementing the TLL is that consumers–who are the main targets of the policy–, have not been adequately and effectively acquainted with it. Although the colorful nature of the TLL is an advantage to consumers, it has created numerous obstacles in the industrial sector. Nutritionists favor the TLL and food industry experts favor the NFL. However, experts from either field do not find nutrition labeling necessary for all packaged products.

## Supporting information

S1 File (DOCX)
