# Supplementary material for: Nutrition labels’ strengths & weaknesses and strategies for improving their use in Iran: A qualitative study
Source: PLoS One. 2020 Oct 30;15(10):e0241395. doi: 10.1371/journal.pone.0241395 (PMC7598474; doi:10.1371/journal.pone.0241395)
Supplement: S1 File — (DOCX) [file pone.0241395.s001.docx]

**STEP I**

**Method: directed content analysis**

**Theme 1: Strengths**

**Theme 2: Weaknesses**

**Theme 3: Strategies to improve the use of nutrition labels**

**STEP II**

**Code system: (mothers) school 1, 2, 3 and 4**

**Theme 1: Strengths [number of frequencies]**

- The right to choose a healthier product [2]
- Inability to pay attention to numbers after seeing colors [1]
- Note the color red [2]
- Color and simplicity [6]
- Impact on children's health if teaching [1]
- Awareness of the amount of energy received [3
- Diet [1]
- The importance of calorie [3]
- The importance of nutritional information [5]
- opportunity [2]
- Fat sensitization [2]
- Disease [5]
- Sensitization by a nutrition consultant [1]
- Health status [8]

**Theme 2: Weaknesses**

- Incomplete label information of some products [2]
- No effect of labels on selection [2]
- Fonts [3]
- Culture [3]
- Not attractive [2]
- Lack of trust in the accuracy of label information [6]
- Large amount of information [2]
- Incompatibility of some tag information [7]
- Information and advertising [6]
- Color [1]
- Small label size [9]

**Theme 3: Strategies**

- Show nutrition information on store shelves [1]
- Culture building [4]
- Increased attention following diseases [1]
- Product packaging coloring [1]
- Encourage factories to produce healthy products [1]
- Information through popular media and programs [14]
- Education [6]
- Supervision of food industry and its publication [1]
- Informing the authorities accepted by the people [1]
- Product sizing by age group [1]
- Verification of labels by regulatory bodies [1]
- Encouraging children in schools [1]
- Information and education in schools [7]
- Simple information [2]
- Fonts [1]
- label location [2]
- eligibility [2]

**Code system (food quality control experts) factory 1**

**Theme 1: Strengths [number of frequencies]**

- The psychological impact of labels [1]
- Can be used for people with certain diseases [1]

**Theme 2: Weaknesses**

- Not considering the age ranges [1]
- Lack of consumer awareness [2]
- Small label area [1]
- Quick implementation of TLL [2]
- Lack of Information and advertising [3]
- Impossibility of reformulation [2]

**Theme 3: Strategies**

- Definition of nutrition barcode [1]
- Coloring based on product quality [1]
- Notification [2]
- Training of TLL appropriate to diseases [1]
- The effect of media on public [1]
- support [2]
- Culture building [2]

**Code system (nutritionists and food industry experts)**

**Theme 1: Strengths [number of frequencies]**

- TLL is better than NFL [4]
- NFL is better for the people with nutrition knowledge[3]

**Theme 2: Weaknesses**

- The difficulty of using numbers in a nutrition facts table[2]
- Incompatibility of the knowledge of the Iranian people with European[1]
- Difficulty in defining portion sizes[2]
- Playing with numbers in the industry[2]

**Theme 3: Strategies**

- Notification [2]
- Culture building [2]
- Brief information [2]

**STEP III: Reorganization of codes**

| **Codes** | ***Mothers*** |
| --- | --- |
| Healthier food choices at the point of purchase | *For example, there are several products in the store; one has 12 grams of fat, the other has 5 g or 8 grams. I read; this one has too much fat. I take that one, however, its taste may be affected a little, but I get used to it. The children got used to it too, for example, it was not possible for us to eat low fat milk and yogurt, but now at home we use low fat dairy products****.*** |
| Reduction in purchase and consumption of high calorie products | *I do not lie; I buy, but try to use less****.*** |
| Easy to understand and prominent red light of the TLL | *We pay attention to traffic light labels when we want to buy. The red line is important, so we consider all of this in our purchases.* |
|  | *Since TLL is shown with colors, it doesn't need much attention. If you are suffering from obesity, or other maladies, you will understand if the item is good for you or not.* |
| Increased thoughtfulness following diseases | *When I want to purchase something, I read the label for the amount of sugar and I make the better choice for myself because I suffer from diabetes.* |
| Labels’ small sizes, fonts and inappropriate location in packaging | *Labels are too small: Sometimes I do not have my glasses with me, or maybe I'm in a hurry to buy something. My eyes cannot see small print. If it is so important, the print should be larger. Now, I am not very old, but the elderly cannot read these small print labels at all.* |
| Mistrust in labels and information provided by manufacturers | *We have lost our trust in manufacturers and we do not believe in their information on the labels.* |
| Unattractive labels | *There are different kinds of labeling that bore consumers when they have to read all of the information.* |
| Ambiguous and high amount of information provided | *There is a great amount of terminology that we do not understand.* |
| Traffic light label (TLL) versus nutrition fact label (NFL) | *TLL is better because it is color-coded. It attracts more attention, and can be more easily understood at a glance. For example, we know green means “good”****.*** |
| Notification via mass media | *It's been sometime that power consumption information has been shown in the middle of TV commercials in the form of A and B labels. When such form of broadcasting increases, I feel that most people who want to buy appliances pay attention to it. My 80-year-old mother has learned this. My 6-year-old son, too, has learned this and when he sees a short flash he knows it means energy consumption is low.* |
|  | *First of all, it should be informative, because if not, it will be another type of broadcast. When it is informative, we will understand.* |
| Community education | *We have not even seen training regarding which (foods) are more suitable for different ages.* |
| **Codes** | ***Food quality control experts*** |
| Colorful | *It is color-coded and somehow compatible with the level of knowledge of people.* |
| Community education | *It should be an education for the whole community. For example, diabetics should not eat biscuits with red meat fat or sugar, but eating 100 grams per day for healthy people will present no problem.* |
| Weight definitions | *According to the national standard, nutrition tables must be based on 30 grams. Our package is 40 grams, so how can I write 30 grams? TLL also has another weight definition, as well. This inconsistency between national standards and the FDA has become a problem for us everywhere.* |
| Impossible to reformulate | *The formula has changed a little in some products. For example, we put a little less salt in a product that has salt on the border of the color definition then the indicator turns* ***from amber to green.*** |
| Failure of authorities to assess factories’ claims | *For example, the competitor uses 30% sugar in their biscuits and the standard definition does not matter to them at all. The color green is also in their TLL. There is no assessment for factories’ claims.* |
| Incompatibility of nutrition labels with public culture | *Iranian consumers, most of whom are ordinary people, do not look at labels and just buy the package****.*** |
|  | *Our point of view does not allow us to do the right thing [read nutrition labels], because we are people of tradition****.*** |
| Financial burden | *We paid too much money for cylinders and wrappers to print TLL.* |
| Possibility of fraud | *They [manufacturers] see their products are turning red, so they might search for ways to bring them to amber. This may result in fraud.* |
| Different coloring responses from different laboratories | *Once, with a very small difference, a product in a laboratory turned amber for a nutrient, however, in another laboratory it turned red.* |
| Inconsistency between policymakers | *If we pour a lesser amount of, for example, sugar in fruit juice it could have the aspects of punishment, violations and profiteering****.*** |
| **Codes** | ***Nutritionists and food industry experts*** |
| Misleading consumers | *We made something ourselves, TLL. Not only does it not guide consumers, but also misleads them.* |
|  | *I am a nutritionist and the first time I saw TLL I did not understand what it meant, even though I am a nutritionist. I was more comfortable with NFL. These TLL colors do not tell me anything.* |
| Ignoring manufacturers' problems and their facilities | *Please listen to what the manufacturers say. You have to value their words and what they are producing, printing, and designing on their labels. You have to conscientiously listen to what they say about the problems they have with this issue.* |
| Following international standards | *Why in the world are we looking to invent the wheel from scratch? There is already an FDA standard table [Codex Alimentarius Standards] with all the specifications you want based on a meal; a 15 gram spoon of food. The whole world follows this pattern.* |
| Different weight definitions | *One item has defined weight in 100 grams, one in 5 grams and yet another in 30 grams. The consumer is confused. For this reason, the producer does not help at all.* |
| Culture-building | *If I am a person who cares about calories, I will buy the product with less calories; it does not matter if it has TLL or NFL labeling. If I have become cultured, it means that I understand that when the label turns red, it means that the item is dangerous.* |
| TLL is easier to understand than NFL | *NFL is very difficult to understand for the society that does not have nutrition knowledge.*  *TLL is a step forward****.*** |
| Importance of food choices | *I think this is the first time that the FDA in Iran has acknowledged that people's food affects diseases. This is a step forward, that is, they have come to realize what we have been saying, that yes, what people eat affects diseases. These are positive points. They have come to understand that salt is important, sugar is important, trans fat is important, and this means that someone has passed this information on to them.* |
| Failure to implement correctly | *The idea is good, but it was not implemented well, because of the resistance we had from the industry, and no one was willing to use red labeling.* |
| **Codes** | ***Policy makers*** |
| TLL guideline by Iran’s FDA | *There is a guideline and I give it to you to read. Everything is clear. When you see the food quality control expert not complying with the guidelines and doing the job the wrong way, it means that the person is not properly trained.* |
| Dairy fats | *Whole milk was given the green label; whole milk which is 3% or 3.5% fat. Since it was the beginning of using nutrition labels, we were very worried. We were worried that our country's dairy consumption would be affected, so at that time they said that we should make all the indicators green in the case of milk, but this can be re-examined.* |
| Education via media | *Unfortunately, the media, especially television, did not cooperate properly with us in this field.* |
| Failure of correct implementation of TLL | *What we did was a giant job; only made a mistake in one or two places, otherwise, it was a great job, however, the government did not support with a budget and we did not have a plan; they just said that it should be done. Fortunately the universities cooperated.* |
|  | *Our labels are badly defined, maybe it is our fault.* |
|  | *We presently have challenges to face. Some industries have concerns and worry about the products that have red labeling.* |
| Controversy surrounding monitoring | *Monitoring has been greatly reduced due to duplication of work, lack of adequate funding, staffing, and accountability.* |

**STEP IV: Classifying codes according to themes (table 2, 3 and 4 of the manuscript)**
